# Buffalo General Hospital

**Published:** 1858-11

**Authors:** 


*Buffalo General Hospital.*—We were gratified by an editorial in the "Peninsular and Independent," on the Buffalo General Hospital, complimenting the profession and citizens of our city on their energy in advancing this enterprize. The part of the hospital which has been erected, is now fitted up for the reception of patients, and has been in operation for some months under the care of Dr. Newman, as Attending Physician, and Dr. Eastman, as Attending Surgeon. The students of the college who are here during the preliminary term, have had opportunities of seeing cases under the guidance of the attending surgeon. We are full of hope that it will not be long before the entire building will be completed, when we will have one of the most perfect hospitals in the State. The position and importance of Buffalo certainly demands such an institution, and the energy of the men engaged in the enterprise leaves no doubt as to its success.

